# Genomic analysis of NE Atlantic sardine (*Sardina pilchardus*) reveals reduced variation in a recently established North Sea population and directs reconsideration of management units

**DOI:** 10.1002/ece3.70101

**Published:** 2024-08-01

**Authors:** Niall J. McKeown, Fabio Campanella, Joana F. Silva, Beatriz A. Roel, Amy J. E. Healey, Paul W. Shaw, Jeroen van der Kooij

**Affiliations:** ^1^ Department of Life Sciences Aberystwyth University Aberystwyth UK; ^2^ CEFAS Lowestoft UK; ^3^ National Research Council (CNR) Institute for Biological Resources and Marine Biotechnologies (IRBIM) Ancona Italy

**Keywords:** fishery, genetic, leading‐edge, pelagic, range shift, stock, sustainability

## Abstract

The European sardine (*Sardina pilchardus*) is under intense fishing pressure and exhibits distributional/abundance shifts linked to environmental change. The current understanding of population demographics needed for sustainable management is uncertain due to concerns that previous genetic studies lacked resolution and limited sampling of sardine north of the Bay of Biscay. To address these issues, we performed mtDNA sequencing and genome wide SNP analysis of samples collected across the Bay of Biscay, Celtic Sea, English Channel and North Sea. The complete SNP data reported a lack of structure throughout the sampled area compatible with high gene flow. A consensus suite of positive outlier SNPs was identified which reported a significant correlation with geographical distance with the largest differentiation between the southern Bay of Biscay and North Sea samples which also reported a significant mtDNA Φ_ST_. While the roles of dispersal limitation and environmental heterogeneity underpinning this require further study, this adds to growing evidence that selection is influencing sardine population structure against a background of high gene flow. The results indicate that while there may be a level of demographic independence between North Sea and South Biscay sardine, the current delimitation of central (Biscay) and northern (Channel and Celtic Sea) operational stocks may misrepresent connectivity between the Biscay and Channel. The North Sea sample exhibited markedly lower mtDNA and nuclear variation than other samples. As sardine have only recently invaded the North Sea such reduced genetic variation is compatible with predictions for peripheral leading‐edge populations but contrasts with patterns for other small pelagic species and emphasises the need to consider species‐specific genetic structure in ecosystem‐based management. Nascent management of the North Sea sardine fishery must ensure that current low levels of genetic diversity are not eroded further as this may decrease the species adaptive potential and inhibit its expansion.

## Graphical abstract

Genomic analysis reveals high gene flow in NE Atlantic sardine which suggests current management units need to be reconsidered. The detection of a small number of outlier SNPs adds to evidence that selection may be influencing sardine populations even in cases of seemingly high gene flow. Reduced genetic variation observed for the North Sea population is compatible with expectations for such leading‐edge populations but contrasts with patterns for co‐distributed small pelagic fishes. This emphasises the need to consider species specific genetic structure in ecosystem‐based management.

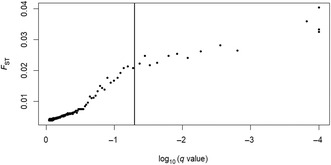

## 1 INTRODUCTION

Small pelagic fish, such as the European sardine, *Sardina pilchardus*, provide approximately half of the worlds annual fish harvest (Freon et al., 2005; Peck et al., 2021). They are a key component of marine ecosystems, serving as a trophic link between plankton and predators (Cury et al., 2000). Most are highly fecund, highly mobile and short lived and some can spawn all year‐round. These characteristics mean they respond quickly to environmental changes; with their sensitivity underpinning marked fluctuation in abundances and altered spatial distributions (Alheit et al., 2012; Alheit & Peck, 2019; van der Kooij et al., 2024). The dramatic stock fluctuations observed for small pelagic fishes have had significant adverse economic consequences for fishing communities and even entire countries, and have profoundly affected ecosystems (Alheit & Peck, 2019). While there are variety of methods to characterise marine populations, not all are suitable for every species and circumstances. For example, tagging may not be possible for smaller fish. Genetic methods can be applied to all organisms and may convey information on population structure and resilience beyond other approaches (Waples et al., 2008) and inform efforts aimed at ensuring fishery sustainability and biodiversity conservation (Kerr et al., 2017; Reiss et al., 2009).

The European sardine (hereafter sardine) is a small pelagic schooling fish distributed in coastal waters of the eastern Atlantic Ocean (from Africa to the North Sea) and in the Mediterranean Sea (Grant & Bowen, 1998). This species has a high dispersal potential, spawning grounds throughout its distribution (Garrido et al., 2016, 2017) and exhibits pronounced variation in population dynamics at both larval, juvenile and adult stages linked to climatic variability (Garrido et al., 2016, 2017; Silva et al., 2008, 2009). It is heavily exploited throughout its range and represents one of the most valued fisheries in the Northeast Atlantic (ICES, 2019). Here, three stocks are described, a Northern stock (Celtic Sea to English Channel), a Central Stock (Bay of Biscay) and a Southern Stock (spanning the Cantabrian Sea to the Gulf of Cadiz) (ICES, 2019). However, the validity of current management boundaries is a matter of debate as there is no consensus as to the species' biological population structure (reviewed in Neves et al., 2021, 2023). The numerous phenotypic based studies (e.g. body morphometrics (Baibai et al., 2012; Mounir et al., 2019); otolith shape (Jemaa et al., 2015; Neves et al., 2021, 2023); otolith microchemistry (Correia et al., 2014)) and genetic studies (e.g. allozymes (Chlaida et al., 2006, 2009; Laurent et al., 2007); mtDNA (Atarhouch et al., 2006; Tinti et al., 2002), microsatellites (Gonzalez & Zardoya, 2007; Kasapidis et al., 2012; Ruggeri et al., 2012, 2013)) have generally provided inconclusive, and at times contradictory, delineations of stock boundaries (Neves et al., 2023). A central issue relates to the fact that genetic studies, the only means to confirm restricted interbreeding, have typically reported weak or absent population structure over wide geographical areas which may mask a diversity of demographic scenarios (Caballero‐Huertas et al., 2022). Furthermore, it is uncertain if the lack of genetic structure reported in sardine reflects realised connectivity or simply limitations in the analytical power provided by small numbers of loci employed in those studies (Caballero‐Huertas et al., 2022; Hellberg, 2009; Waples et al., 2008).

The uncertainties as to population structure of sardine are particularly important given that catches in some regions have declined, biomass estimates have fluctuated significantly, and some stocks have been recognised as fully exploited in recent times (ICES, 2017, 2019, 2021). Furthermore, climate change is changing the distribution of fish populations by different mechanisms including direct displacement of populations into novel areas and increased productivity of peripheral populations (Petitgas et al., 2012). Such processes are evident for sardine in the North Sea which has become warmer in recent decades as a result of climate change (Perry et al., 2005). Sardine is considered to have re‐invaded the North Sea in the 1990s, and there is now an established spawning population in the Northern shelf area (Alheit et al., 2012). The nature of this expansion is uncertain as no genetic studies, except for Kasapidis et al. (2012), have included samples collected north of the Bay of Biscay. More broadly, few studies have dealt with the effect of climate change on the genetic variation of commercially exploited fish species specifically (Crozier & Hutchings, 2014).

Genome wide SNP analysis has provided enhanced resolution of marine population structure and local adaptation beyond that of methods surveying smaller numbers of loci. As such it has proven to be a useful tool in assessing the validity of fishery management boundaries (Leone et al., 2019; Mullins et al., 2018). The recent genomic study by Antoniou et al. (2023) showcased the potential of genomic approaches in sardine by reporting robust genetic divergence of Mediterranean samples from a sample collected in the Gulf of Cadiz (Atlantic). Accordingly, the primary objective of this study was to investigate sardine population structure among the Biscay, Celtic Sea, English Channel and North Sea regions (hereafter termed NE Atlantic) using RAD‐seq. The rationale being that the ability to survey large numbers of loci, and potentially identify outlier loci, would permit a powerful test of the congruence between the current management units and biological populations (Reiss et al., 2009). The inclusion of a North Sea sample also permitted assessment of genetic composition in what could be considered a leading‐edge population in comparison to more central populations (Biscay‐English Channel). Based on results for other small pelagic species, we predicted that levels of genetic variation would be broadly similar across samples owing to the seeming ability of these species to rapidly track suitable habitats in large numbers (Silva et al., 2014). The SNP analysis was complemented by mtDNA sequencing to permit comparison of phylogeographic patterns with other co‐distributed pelagic fishes (Debes et al., 2008; Silva et al., 2014; Zarraonaindia et al., 2012).

## 2 MATERIALS AND METHODS

### 2.1 Sample collection and DNA extraction

**FIGURE 1:**
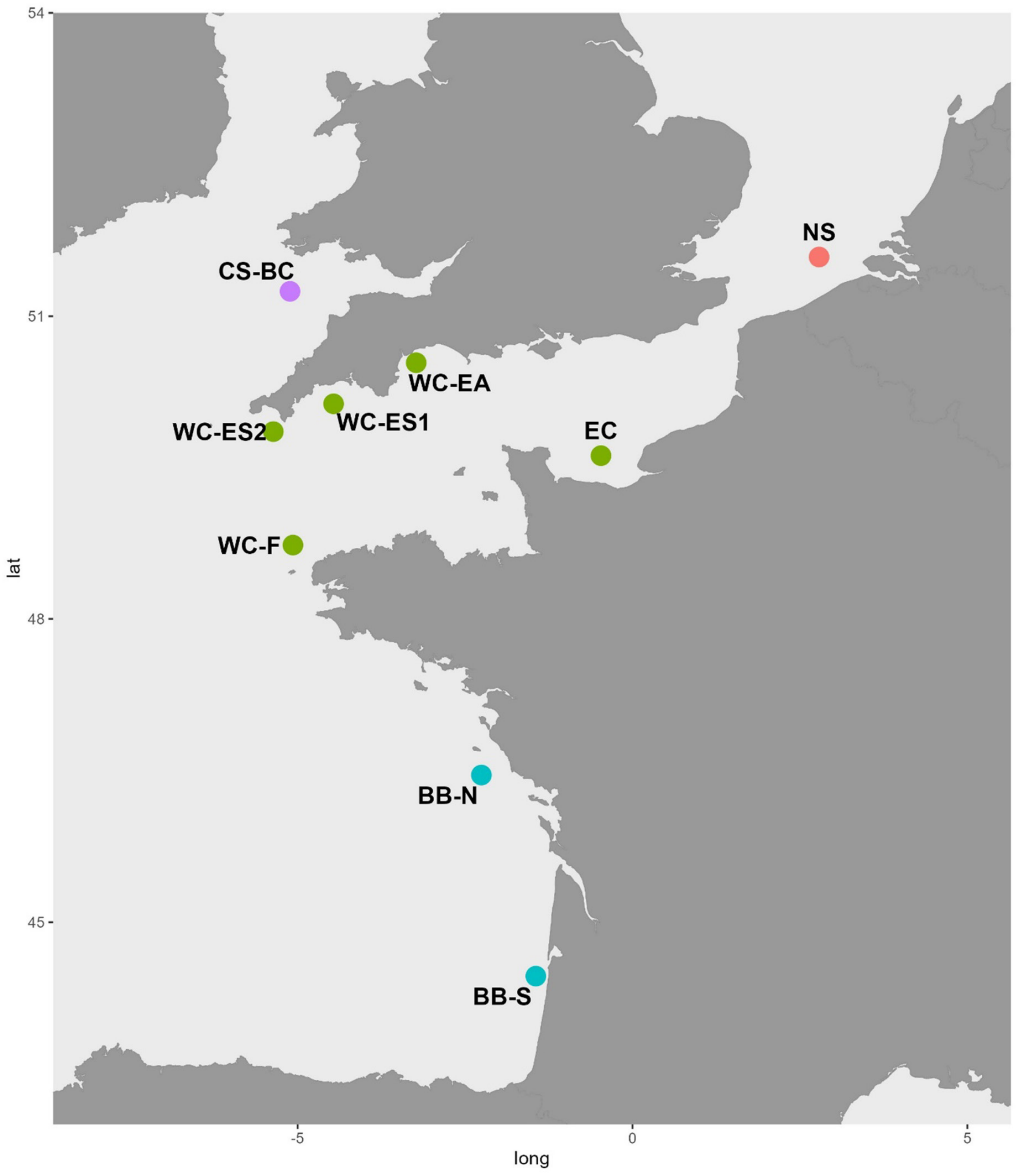
Location of sardine samples processed in this study. Sample labels correspond to Table 1 which provides additional details.

Samples of adult sardine were collected from 9 sites across the Bay of Biscay, English Channel, Celtic Sea, and North Sea, by means of research vessel surveys and fishing vessels (Figure 1, Table 1). Samples were processed and fin clips preserved in ethanol until DNA extraction was performed using a QIAGEN DNeasy Blood & Tissue kit following manufacturer's instructions. Hereafter, the term “sample” denotes a collection of fish from a specific location.

**TABLE 1:** Sample information and summary indices of genetic variation for mtDNA and the complete nuclear (all SNPs) and outlier SNP datasets.

Sample site *survey*	Code	Lat:Long *date*	Sample size (mt:Nu)	mtDNA		Nuclear: All SNPs				Nuclear: Outliers			
				H (SD) *Nhap*	Tajima's *D* Fu's *F*'s	N poly	*H*_O_	*H*_E_	*F*_IS_	N poly	*H*_O_	*H*_E_	*F*_IS_
Bay of Biscay (South) *PELGAS10*	BB‐S	44.4661: −1.44377 *29/04/10*	24:30	0.714 (0.102) *11*	−2.166 −9.274	2367	0.302	0.331	0.08	14	0.439	0.397	−0.10
Bay of Biscay (North) *PELGAS10*	BB‐N	46.45521 −2.25638 *06/05/10*	22:30	0.601 (0.121) *8*	−2.129 −5.234	2361	0.301	0.326	0.07	14	0.392	0.403	0.02
Western English Channel (French) *PELTIC10*	WC‐F	48.1926 −5.07003 *15/06/10*	32:27	0.613 (0.101) *11*	−2.108 −9.004	2263	0.285	0.321	0.11	13	0.331	0.353	0.07
Western English Channel (English) *Industry*	WC‐ES2	50.134 −4.46324 *1/7/10*	25:30	0.543 (0.118) *8*	−2.035 −6.629	2371	0.309	0.332	0.06	14	0.229	0.302	**0.24**
Western English Channel (English) *PELTIC17*	WC‐ES1	49.8592 −5.36325 *20/10/17*	24:30	0.565 (0.121) *9*	−2.281 −5.653	2366	0.306	0.327	0.06	14	0.404	0.396	−0.02
Western English Channel (French) PELTIC17	WC‐EA	50.84018 −3.22983 *28/10/17*	29:32	0.657 (0.099) *10*	−2.021 −8.377	2371	0.301	0.329	0.08	14	0.329	0.379	0.13
Celtic Sea (Bristol Channel) *PELTIC17*	CS‐BC	49.6214 −0.471 *3/10/17*	30:31	0.561 (O.101) *10*	−2.152 −7.446	2324	0.282	0.324	0.13	14	0.311	0.321	0.03
Eastern English Channel *PELTIC18*	EC	51.24653 −5.11378 *8/11/18*	32:32	0.643 (0.094) *11*	−2.273 −8.552	2368	0.301	0.331	0.09	13	0.312	0.306	−0.02
North Sea *IBTS2016*	NS	51.586 2.78533 *8/8/16*	29:39	0.320 (0.117) *6*	−2.01 −5.399	1565	0.238	0.315	**0.24**	12	0.211	0.248	**0.16**

*Note*: mtDNA variation reported includes haplotype diversity (*H*) and number of haplotypes (Nhap) and Tajima's *D* and Fu's *F*'s tests. Nuclear variation is described using number of polymorphic loci (Npoly), observed and expected heterozygosities (*H*_O_ and *H*_E_, respectively) and *F*_IS_ (with significant values in bold).

### 2.2 Mitochondrial DNA analysis

A 365 bp portion of the mtDNA cytochrome B gene was amplified by PCR in a subset of randomly selected individuals from each sample using the primers: forward 5′‐ CGAACATCTCGGTCTGATGA‐3′ and reverse 5′‐ GACGGCGGACAGTAGGTTAG‐3′. PCRs were performed in 10 μL volumes containing ~50 ng template DNA, 5 μL Biomix (Bioline UK), 0.025 UM of each primer and using a thermoprofile consisting of an initial denaturation step (95°C for 3 min) followed by 35 cycles of 95°C for 30 s, 50°C for 30 s and 72°C for 30 s, and a final cool down step (4°C for 60 s). Amplicons were sequenced with the reverse primer using BigDye technology on an ABI 3500 system (Applied Biosystems) following manufacturer's recommendations. Sequences were edited using CHROMAS and aligned using BIOEDIT (Hall, 1999). mtDNA data were analysed in ARLEQUIN (Excoffier & Lischer, 2010) unless stated otherwise. Genetic variation was estimated using haplotype (h) and nucleotide (p) diversity. A minimum spanning network was constructed in NETWORK (www.fluxus‐engineering.com/sharenet.htm). Differentiation between pairs of samples was quantified using pairwise Φ_ST_ with significance assessed by 10,000 permutations. Fu's *F*'s (Fu, 1997) and Tajima's *D* (Tajima, 1989) were used to test for deviations from mutation‐drift equilibrium. Mismatch distributions, the frequency distributions of pairwise differences between haplotypes within a sample and simulated distributions under a model of demographic expansion, were compared using the sum of squared deviations (SSD) as a test statistic with significance assessed after 10,000 bootstrap replications.

### 2.3 Genome wide SNP analysis

SNP genotyping by sequencing was performed using tunable Genotyping By Sequencing (tGBS), a modified version of RAD‐seq that incorporates digestion with two enzymes for genome reduction and results in an increased number of reads per site (Ott et al., 2017). Briefly, genomic DNA was digested with the restriction enzymes: NspI and BfuCI/Sau3AI leaving 3′ and 5′ overhangs, respectively, to which single‐stranded adaptor oligos were ligated. Following PCR, the tGBS libraries were sequenced on a Life Technologies' Ion Proton System. Sequenced reads were analysed using a custom Perl script (available at https://github.com/orgs/schnablelab), which assigned each read to a sample and removed barcode sequences. Seqclean (sourceforge.net/projects/seqclean) was used to remove proton adaptor sequences and chimeric reads harbouring internal restriction enzyme sites. Retained reads were subjected to quality trimming in two phases using the software Lucy2 (Li & Chou, 2004) in which bases with PHRED scores <15 (of 40) were removed. In the second phase, the remaining sequences were scanned using overlapping 10 bp windows. Trimmed sequence reads from all samples were combined and normalised to a maximum of 100× coverage, using diginorm (Brown et al., 2012). Trimmed reads were aligned to the reference genome using GSNAP (Wu & Nacu, 2010), and reads were filtered if mapped uniquely. A SNP was called homozygous in an individual if at least 15 reads supported the genotype at the site and at least 90% of all reads covering that site shared the same nucleotide. A SNP was considered heterozygous in an individual if each of the two nucleotide variants were reported at least 15 times, and each allele was represented in more than 35% of the total reads. As part of further filtering polymorphisms in the first and last 3 bp of each quality trimmed read were ignored and each retained polymorphic based must have a PHRED base quality value >20. As a final step, data were further filtered to generate two data sets wherein the minimum allele frequency (MAF) was set at 10% and 1%. Following recommendations by Roesti et al. (2012) we report the results for the MAF = 10% dataset however the corresponding results for analysis of the MAF = 1% dataset can be found in the supplementary material and revealed identical patterns.

ARLEQUIN was used to estimate allele frequencies, observed (*H*_O_) and expected (*H*_E_) heterozygosity, and to test for departures from expectations of Hardy–Weinberg Equilibrium (HWE) and calculate *F*_IS_. Genetic differentiation among samples was quantified by global and pairwise *F*_ST_ (Weir & Cockerham, 1984) with statistical significances evaluated in ARLEQUIN with 10,000 permutations and a missing data threshold of 0.1 per locus. GENALEX (Peakall & Smouse, 2006) was used to perform a principal co‐ordinate analysis of pairwise *F*_ST_ values between samples. The Bayesian clustering method in STRUCTURE 2.3.4 (Pritchard et al., 2000) was also employed to (i) identify the most probable number of genetically distinct groups (K) represented by the data and (ii) estimate assignment probabilities (Q) for each individual (specifically their genomic components) to these groups. The analysis was performed with and without the LOCPRIOR model, in both cases assuming admixture. Simulations were run 10 times for each proposed value of K (1–5; higher values of K were tested in shorter pilot runs) to assess convergence. Each run had a burn‐in of 100,000 Markov Chain Monte Carlo (MCMC) samples followed by 1,000,000 MCMC repetitions. Models were assessed using L(K) (Pritchard et al., 2000).

Outlier loci potentially under divergent selection were identified using the independent approaches implemented in BAYESCAN 2.0 (Foll & Gaggiotti, 2008) and the hierarchical FDIST test in ARLEQUIN (Excoffier & Lischer, 2010), and the PCA‐based method in Pcadapt (Luu et al., 2016). For the BAYESCAN analysis, all parameters that could be modified were left as default and the false discovery rate was set at 5% meaning that a marker with a *q* value lower than 0.05 was considered an outlier. In the FIDST analysis loci with significantly higher *F*_ST_ values were considered outliers. For both the BAYESCAN and FDIST analyses, which are based on island models, samples were grouped according to their sample location. For the Pcadapt analysis we first assessed the optimal *K* value (i.e. the optimal number of genetic groups), from 1 to 20, using a scree plot of the proportion of variance explained by each principal component. We then retained *K* = 2 using the method described in the tutorial and generated a list of outliers with an expected FDR of 0.05 following Bonferroni correction which is the most conservative option. Only SNPs identified as deviating from neutral patterns by all three methods were retained as outliers for subsequent analyses. The stringent parameters and consensus approach to outlier identification were employed to account for potential false positives (Ahrens et al., 2018; Narum & Hess, 2011). Functional significance of outlier loci was investigated by analysing the SNP containing sequences using BLAST following Milano et al. (2014).

## 3 RESULTS

### 3.1 mtDNA variation

**FIGURE 2:**
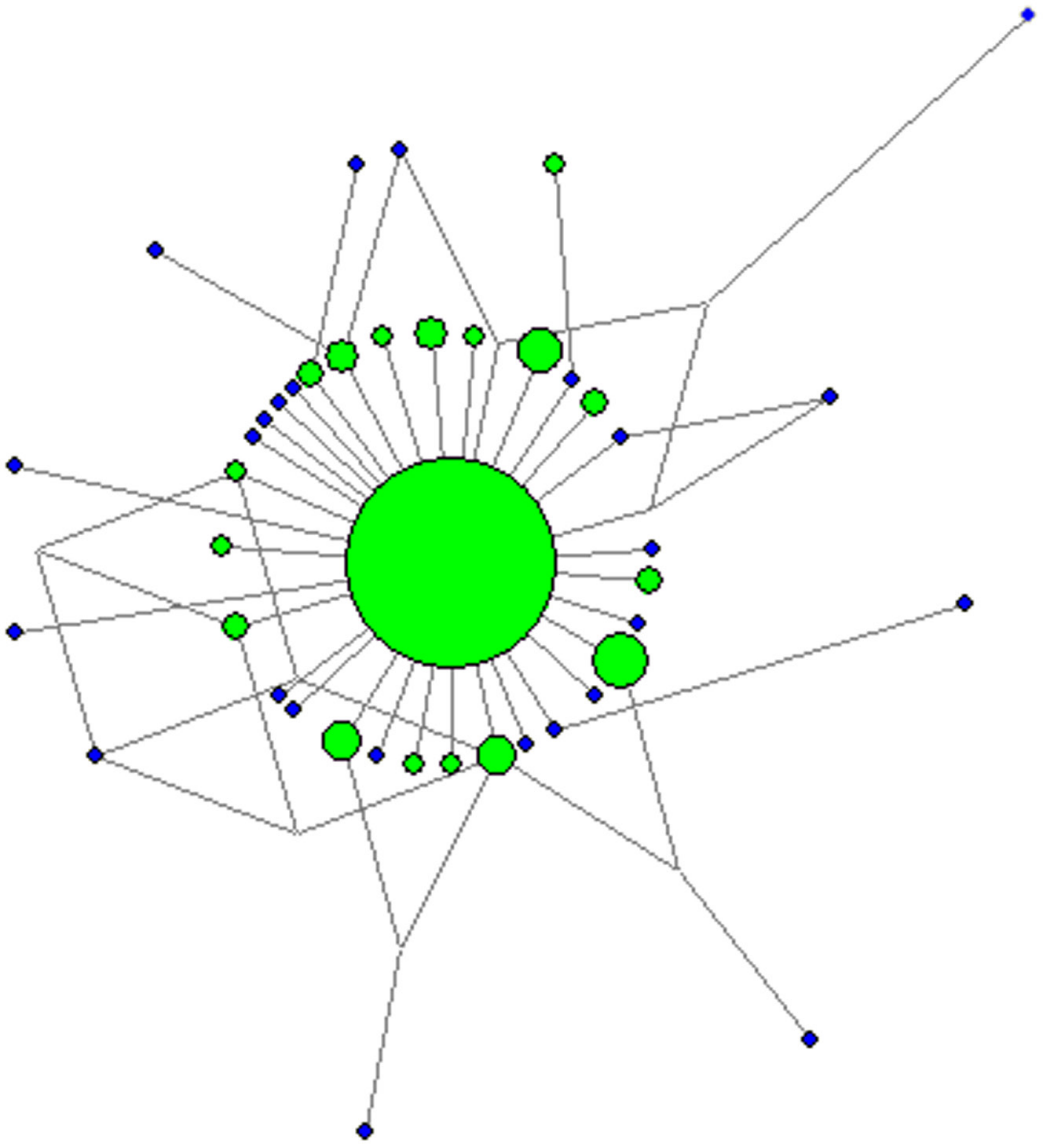
Phylogenetic relationships among haplotypes resolved in this study inferred using a median joining network. Disc size reflects overall abundance and line length is proportional to divergence between sequences. Green discs denote haplotypes found in more than one individual while blue discs denote haplotypes found in a single individual.

The final sequence alignment comprised 331 bp across 247 individuals and revealed 43 haplotypes (overall *H* = 0.605). One haplotype (H 4) was clearly dominant (overall frequency = 0.64), and the most common in all samples. Of the remaining haplotypes only 15 were found in more than one sample site, with the other 27 being found in only one sample site (and typically one individual). Phylogenetic network construction revealed a shallow phylogeny with a classic star‐shaped pattern wherein H 4 was at the centre and most other haplotypes radiated from this with a small number of substitutions (Figure 2).

Summary indices of variation are reported in Table 1. While these revealed broadly similar levels of variation among most samples the North Sea sample exhibited lower *H* and number of haplotypes than the other samples. All samples exhibited significant negative vales for Tajima's *D* and Fu's *F*'s. Mismatch distribution analysis also revealed non‐significant deviation from a model of population expansion for all samples. Pairwise Φ_ST_ values were non‐significant in all cases except the pairwise tests between the North Sea and the South Biscay (Φ_ST_ = 0.065; *p* = .02), and North Sea with the Bristol Channel (Φ_ST_ = 0.049; *p* = .04).

### 3.2 Nuclear variation

Across all 288 individuals genotyped by RAD‐Seq 729,670,646 sequence reads were obtained with an average of 2,533,578 per individual (Average read length = 107 bp; minimum read length = 30 bp; Maximum read length = 236 bp). Filtering to include only SNPs genotyped in 90% of individuals and with a minimum allele frequency of 0.1 resulted in 9882 biallelic SNPs. Further filtering to include only SNPs with a MAF > 0.1 resulted in 2375 SNPs.

Summary indices of variation are reported in Table 1 and reveal the North Sea to exhibit a lower number of polymorphic loci and observed heterozygosity compared to the other samples, which all reported broadly similar values in these cases. The North Sea sample was also the only sample to exhibit a significant multiloci deviation from HWE and positive *F*_IS_ value. Global *F*_ST_ was 0.002 and not statistically significant (*p* = .54). Similarly, all pairwise *F*_ST_ values were numerically small (max value = 0.004 between South Biscay and Eastern English Channel samples) and non‐significant (Table 2). STRUCTURE analysis unanimously supported a value of *K* = 1.

**TABLE 2:** Pairwise *F*_ST_ values with significant values in bold. Below diagonal are values estimated from all SNPs. Above diagonal are values estimated from the 14 positive outliers.

	BB‐S	BB‐N	WC‐F	WC‐ES2	WC‐ES1	WC‐EA	EC	CS‐BC	NS
BB‐S	–	**0.021**	**0.070**	**0.087**	**0.021**	**0.037**	**0.107**	**0.072**	**0.148**
BB‐N	0.001	–	**0.032**	**0.028**	0.007	0.014	**0.060**	**0.031**	**0.087**
WC‐F	0.001	0.001	–	**0.023**	**0.040**	0.011	0.008	0.008	0.021
WC‐ES2	**0.003**	0.001	0	–	**0.035**	0.022	**0.035**	0.014	**0.043**
WC‐ES1	0.001	0.001	0.003	0.002	–	0.018	**0.065**	**0.037**	**0.094**
WC‐EA	0.001	0.001	0.001	0.002	0.001	–	**0.029**	0.010	**0.049**
EC	**0.005**	**0.003**	0.002	0.002	**0.003**	0.001	–	0.020	0.010
CS‐BC	**0.003**	0.002	0.001	0.002	0.002	0.001	0.001	–	**0.027**
NS	0.001	0.001	0.001	0.001	0.001	0.001	0.001	0.001	–

**FIGURE 3:**
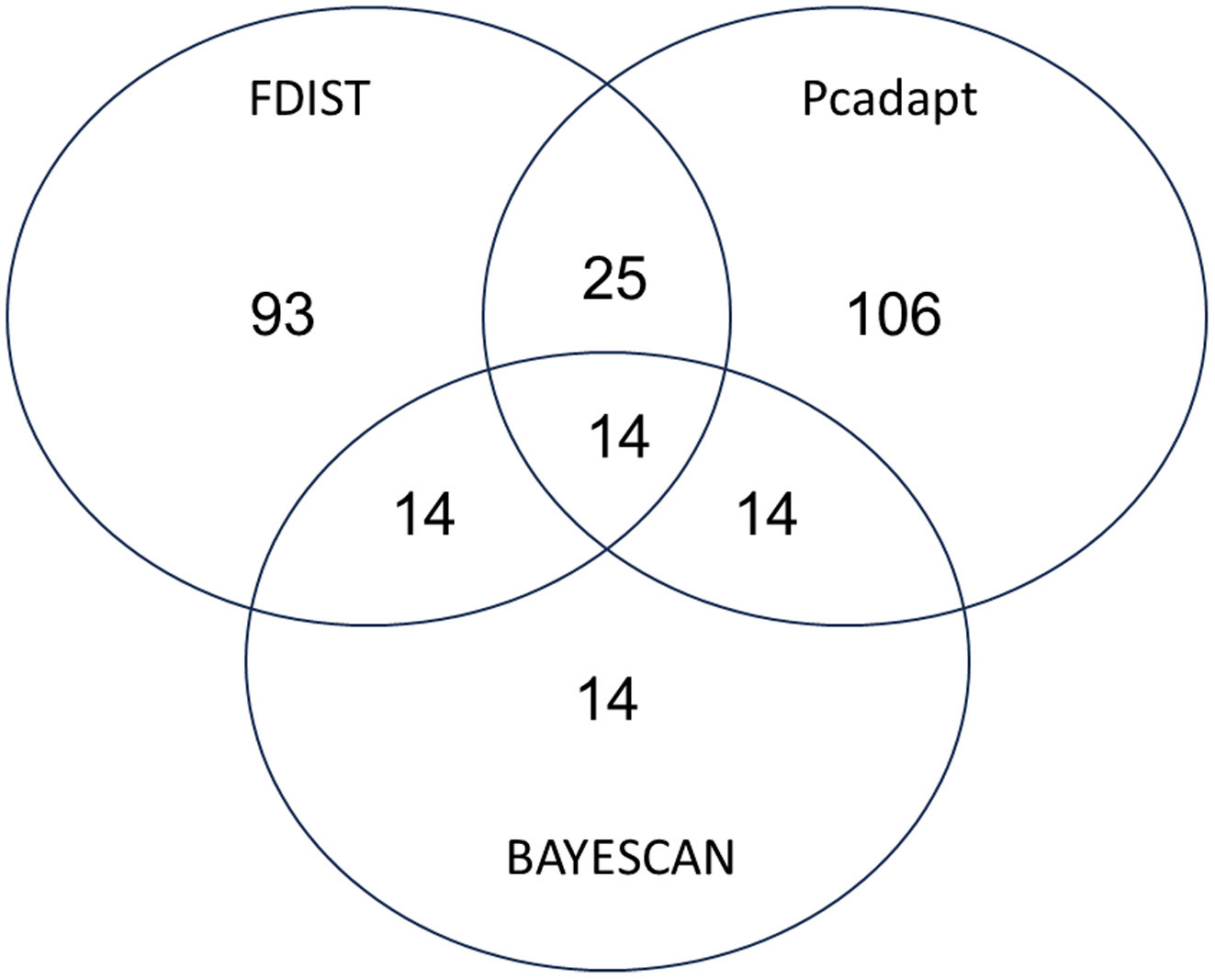
Venn diagram showing the number of outlier SNPs identified by the various outlier detection methods and the overlap between these methods.

**FIGURE 4:**
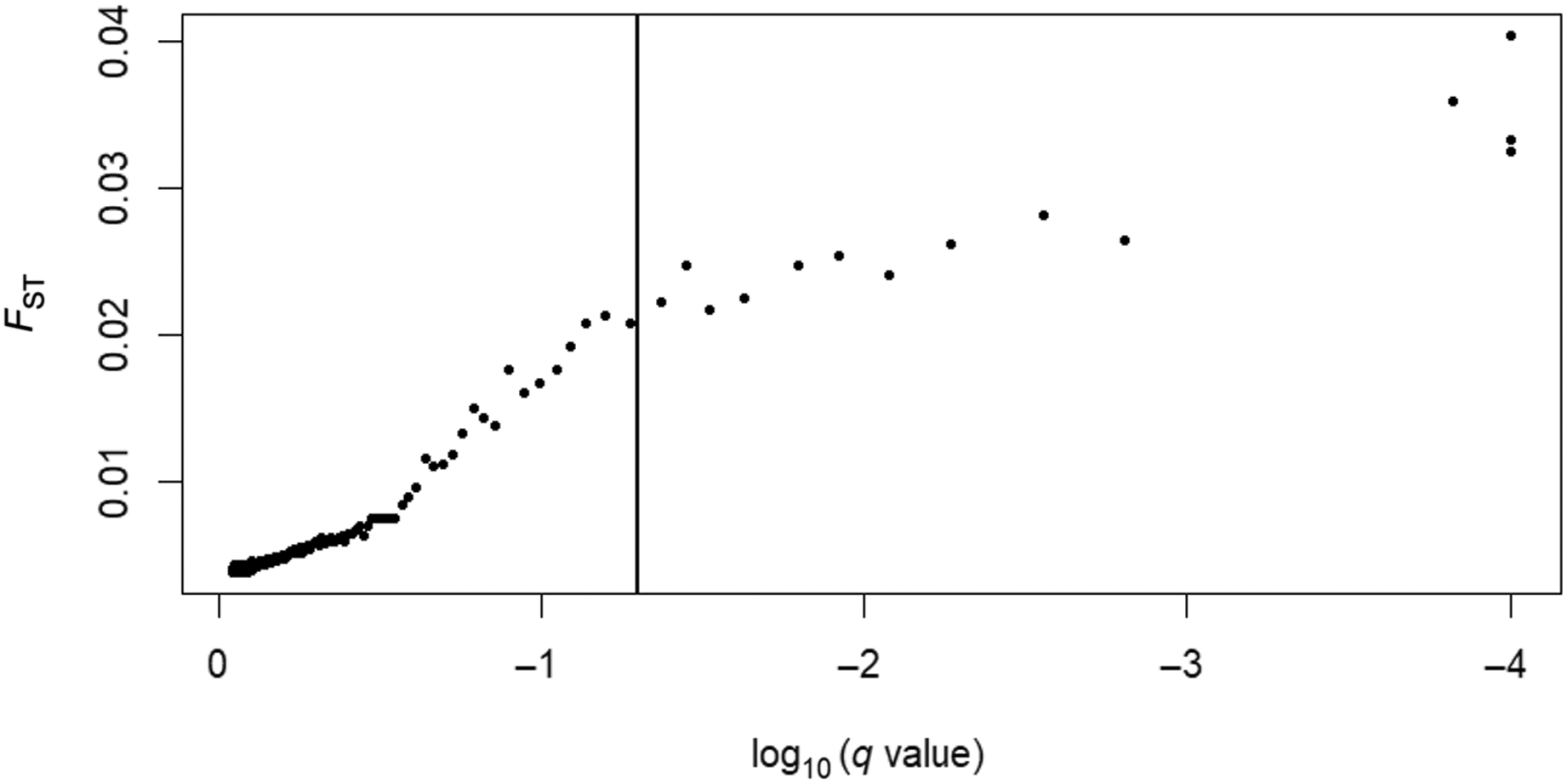
BAYESCAN plot showing the detection of 14 positive outlier SNPs (to the right of the threshold).

The FDIST outlier analysis identified 93 positive outliers. Further inspection revealed 17 positive outliers at the more stringent threshold of *p* < .001. Pcadapt reported 106 outliers, 25 of which overlapped with those identified by FDIST (Figure 3). Fourteen of these 25 corresponded to outliers identified at the *p* < .001 threshold by FDIST. Finally, BAYESCAN identified 14 outliers (Figure 4), all of which had been identified by both the FDIST and Pcadapt analyses (Figure 3). These 14 outliers were considered for downstream analysis. Six out of the 14 outlier SNP containing sequences yielded significant BLAST results which indicated high similarity to mRNA sequences in other fish species (Table 3).

**TABLE 3:** Summary information for the subset of outlier SNPs that revealed significant BLAST results.

Outlier SNP id	BLAST hit	% identity	NCBI accession
Sardine_1011279‐8	PREDICTED: *Clupea harengus* nuclear prelamin A recognition factor‐like (narfl), transcript variant X2, mRNA	89	XM_012818361.2
Sardine_1105148‐93	PREDICTED: *Alosa sapidissima* nuclear factor I/C (nfic), transcript variant X5, mRNA	95	XM_042111921.1
Sardine_1211086‐112	PREDICTED: *Alosa alosa* macrophage‐expressed gene 1 protein‐like (LOC125294218), transcript variant X2, mRNA	100	XM_048242690.1
Sardine_159748‐50	PREDICTED: *Alosa alosa* collectrin‐like (LOC125300033), mRNA	95	XM_048251506.1
Sardine_684899‐15	PREDICTED: *Alosa alosa* BCL6A transcription repressor a (bcl6aa), transcript variant X5, mRNA	95	XM_048252268.1

**FIGURE 5:**
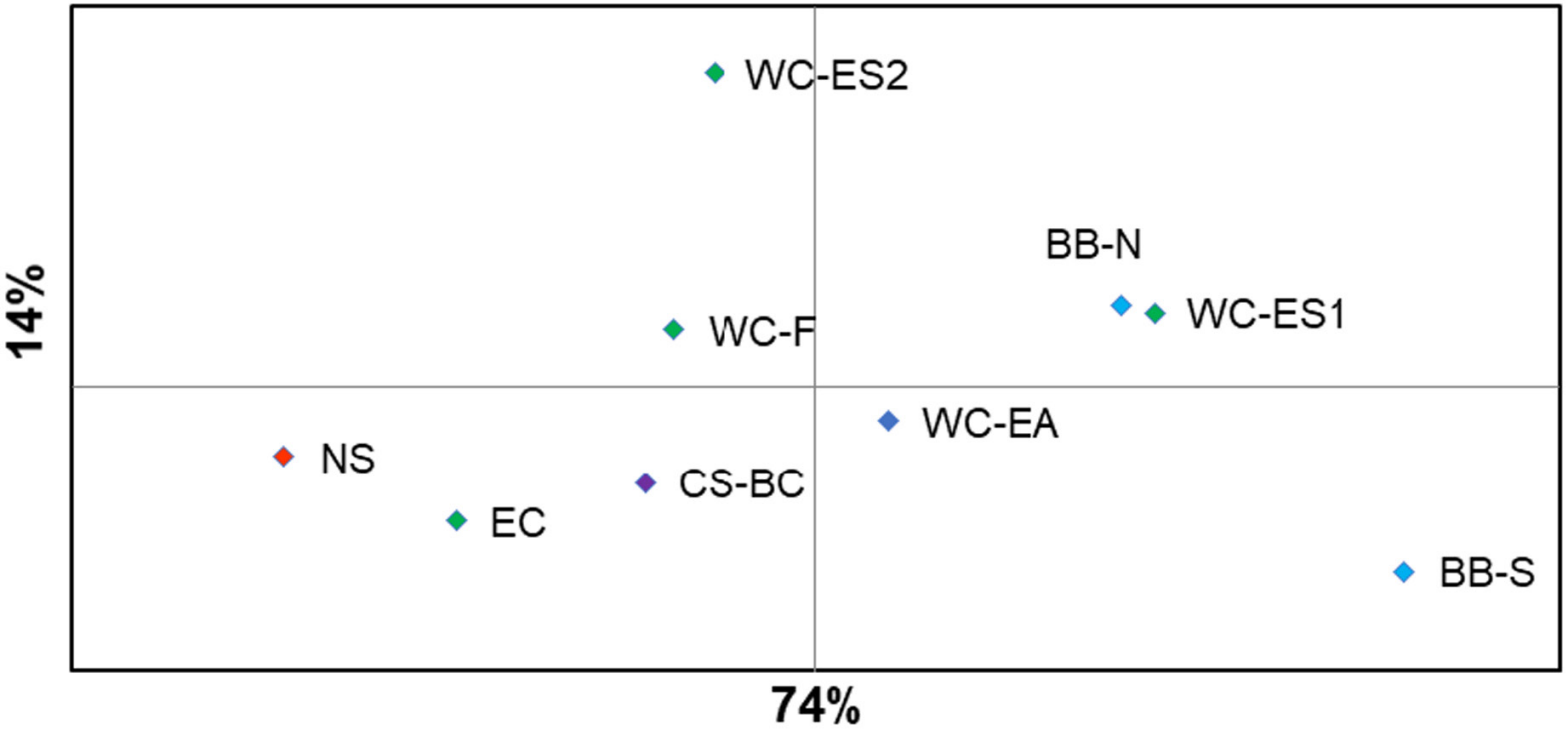
PCoA of pairwise *F*_ST_ among samples based on 14 outlier loci. Sample codes and colour correspond to Table 1 and Figure 1, respectively.

**FIGURE 6:**
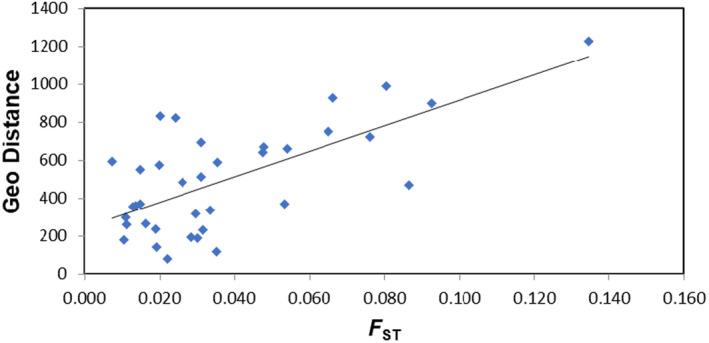
Mantel tests showing the significant correlation between *F*_ST_ (*x* axis) and geographical distance (*Y* axis) based on the 14 outlier SNPs identified among NE Atlantic samples.

STRUCTURE analysis of the outlier genotypes supported an optimal model of *K* = 1. Repeating the analysis at higher *K* values reported no evidence of assignment of individuals to different clusters. However, global F_ST_ based on the outliers was 0.064 and statistically significant (*p* < .001). The largest pairwise F_ST_ was found between the south Biscay and North Sea sample (*F*_ST_ = 0.148; Table 2). Both these samples exhibited large numbers of significant pairwise test results from all other samples. The south Biscay sample was found to be differentiated from all other samples (average *F*_ST_ = 0.059), including the northern Biscay sample. The North Sea sample also exhibited significant *F*_ST_ values in comparison with all other samples except for the East Channel (the geographically closest sample) and West Channel (French) samples (Table 2). The separation between the North Sea and South Biscay samples was also evident from PCoA of *F*_ST_ (Figure 5) and contributed to an overall significant correlation with geographical distance (*R* ^2^ = 0.46, *p* = .01; Figure 6).

## 4 DISCUSSION

This study combined mtDNA sequencing and nuclear genome wide SNP analysis to investigate population structure among sardine collected from the Bay of Biscay, English Channel, Celtic and North Seas. The salient features of the data were (i) a general lack of structure based on the entire SNP data set and mtDNA (ii) identification of a consensus suite of positive outlier SNPs across different neutrality tests which reported statistically significant and spatially coherent F_ST_ values and (iii) markedly lower levels of mtDNA and nuclear (both neutral and non‐neutral) variation for the North Sea population. These findings are interpreted in the context of neutral and adaptive processes shaping sardine populations in the area, including the expansion in the North Sea, and the validity of current management units.

Analyses performed including all nuclear loci reported non‐significant values for global *F*_ST_ and all pairwise *F*_ST_ while individual‐based analyses revealed no evidence of clustering among individuals. This overall lack of nuclear differentiation is compatible with high gene flow across this area. Weak genetic structure has been reported in this region for other fish (e.g. flounder (*Platichthys flesus*), Hemmer‐Hansen et al. (2007); sea bass (*Dicentrarchus labrax*), Souche et al. (2015)) including small pelagic fish (herring (*Clupea harnegus*), Mariani et al. (2005); sprat (*Sprattus sprattus*), McKeown et al. (2020)). Wide ranging gene flow also fits with what is known about ecology and distribution of sardine in the NE Atlantic and potential for both larval and adult‐mediated gene flow. The species is continuously distributed throughout the area and otolith analyses suggest movement of adults among contiguous area (Neves et al., 2023) while ichthyoplankton monitoring has revealed considerable overlap in spawning activity throughout the NE Atlantic (Stratoudakis et al., 2007). The lack of genetic structure may also be linked to historical processes. MtDNA reported a number of signals (star shaped phylogeny, unimodal mismatch distribution, significant *F*'s and Tajima's *D* values) that can be attributed to population size changes and range expansion events associated with the historical colonisation of these waters at various times following the Last Glacial Maximum, as suggested for anchovy (*Engraulis encrasicolus*) (Zarraonaindia et al., 2012) and sprat (Debes et al., 2008) in the NE Atlantic. Such processes mean that the sardine populations are almost certainly not at genetic equilibrium (Hutchinson & Templeton, 1999) and estimates of genetic structure may be biased low owing to retention of signals of historical gene flow.

### 4.1 Detection of outlier SNPS

Genome wide SNP analysis offers the potential to genotype markers that are influenced by selection (outliers) and can reveal differentiation where neutral markers remain uninformative (Allendorf et al., 2010). A common criticism of outlier detection methods is their high rate of false positives and applicability under different demographic scenarios (Lotterhos & Whitlock, 2014). To address this, we employed a recommended consensus approach whereby loci were only considered outliers if identified by three different methods (De Villemereuil et al., 2014; Whitlock & Lotterhos, 2015). It is common that different outlier methods will often report different numbers of outliers with varied levels of overlap (Benestan et al., 2016; Thomas et al., 2017), as various tests have different strengths and weaknesses. Interestingly both the Pcadapt and FDIST methods identified much a larger number of outliers than the Bayescan but while there was limited overlap between the FDIST and Pcadapt outliers, all 14 outliers by BAYESCAN were among those identified by the other two approaches. These patterns fit with previous assessments of the various outlier detection methods. For example, while the Pcadapt method has been shown to perform better than island‐based methods under scenarios of Isolation by distance and range expansion it is known to exhibit increased false positives relative to the island model (Lotterhos & Whitlock, 2014) while high false positive rates have also been reported for the FDIST method (Lotterhos & Whitlock, 2014). In contrast the low number of outliers identified by BAYESCAN and the overlap with the other methods is compatible with the low rate of false positives reported for this approach under a range of demographic scenarios (De Mita et al., 2013; Narum & Hess, 2011). Based on these various test attributes we therefore consider our consensus suite of outliers as robust. Interestingly, Antoniou et al. (2023) did not report agreement upon different outlier detection methods which they propose as being reflective of complex selection processes. Previous studies of sardine based on much smaller numbers of loci have also identified, in some cases incidentally, signatures compatible with selection in the form of elevated divergence at allozyme (Chlaida et al. 2009; Laurent et al., 2007) and microsatellite (Kasapidis et al., 2012) loci. Therefore, we consider the identification of outlier SNPs under the stringent conditions here, with some reporting associations with functional genomic regions, as adding to the growing body of evidence that selection is acting against a background of high gene flow across the species' range.

Although the number of outlier SNPs identified here was small (*n* = 14) and did not permit robust assignment, the corresponding F_ST_ values did show a clear spatial pattern including a correlation with geographical distance and the largest difference between the North Sea and South Biscay samples. This could reflect spatially varying selection. Antoniou et al. (2023) reported that the number of days with sea surface temperature above 19°C (critical threshold for successful spawning sensu Stratoudakis et al. (2007)) was a prominent driver of the clinal genetic structure at neutral and non‐neutral SNPs across the Mediterranean—Cantabrian Sea. Sardine spawning seasonality in Atlantic European waters also seems to be mainly driven by water temperature (Stratoudakis et al., 2007). In the Channel there is a clear double peak in spawning activity with the main periods in Spring–Summer and again in Autumn (Coombs et al., 2010). Moving to lower latitudes the spawning activities are found to be earlier and shorter. Stratoudakis et al. (2007) suggest that there may be a genetic (adaptive) basis to upper temperature tolerance to spawning and that sardine spawning tolerance and preference may clinally vary over the Atlantic distribution. Spatially varying selection is not the only selective regime that may generate outliers. Outliers may be generated by endogenous selection and genomic incompatibilities rather than environmental selection per se (Bierne et al., 2011). As such incompatibilities are typically associated with biogeographic breaks and/or secondary contact between formerly isolated units we consider this unlikely to be a prominent driver of the pattern report here given the geographical region under study and shallow phylogeographic structure resolved by mtDNA. Signals similar to spatially varying selection may also be generated by the spread of advantageous alleles driving clinal patterns with lower diversity among geographical regions near the source of the advantageous alleles (Bierne, 2010). Interestingly variation at outlier loci have been observed to conform to geographical clines in other studies of sardine in NE Atlantic waters. The range wide study by Kasapidis et al. (2012) reported signals of structuring at the microsatellite locus SP22 which was similar to patterns at the allozyme locus (SOD*) and contrasted with the general lack of structure reported at other microsatellite loci analysed. Chlaida et al. (2009) also reported elevated and geographically congruent structuring along the Moroccan coast at the SOD* locus with other allozyme reporting a general lack of structure. Laurent et al. (2007) described a genetic cline at two allozyme loci (PGM‐1* and PEP‐it*) in the Bay of Biscay which they attributed to selection. While the relative roles of allele sweeps and spatially varying selection can not be distinguished at this stage, the additional evidence of clinal patterns of non‐neutral structure might indicate that the patterns observed here are a small segment of a larger cline of non‐neutral structure. A multispecies genetic cline driven by sea temperature minima has been reported in the NW Atlantic (Stanley et al., 2018).

### 4.2 Lower genetic variation in the North Sea

Analysis of genetic patterns in peripheral, leading, or rear‐edge populations can provide information as to how species have responded to environmental changes over different time frames. Studies of sprat (Debes et al., 2008; Limborg et al., 2009, 2012; McKeown et al., 2020) and anchovy (Huret et al., 2020; Silva et al., 2014; Zarraonaindia et al., 2012) have typically reported similar levels of intraspecific genetic variation throughout both species NE Atlantic ranges (excluding the Baltic Sea in the case of sprat). This has broadly been interpreted as a result of post glacial colonisations following the LGM involving large numbers of individuals such that the northern populations retain similar levels of genetic diversity as their southern counterparts (Silva et al., 2014). The similar levels of variability among sardine samples from Biscay to Channel is compatible with such models of little loss of genetic variability during colonisation of this area. In this context, the lower levels of both mtDNA and nuclear (neutral and non‐neutral) for the North Sea sample is striking. Limborg et al. (2012) proposed that reduced variation in marginal Baltic, Adriatic and Black Sea sprat populations could be explained by a combination of founder effects during a recent colonisation and a steppingstone model with lower migration rates and higher genetic drift. Excoffier and Ray (2008) also outline how allelic surfing during colonisation may contribute to reduced diversity. In addition to such neutral (genome wide) processes, non‐neutral processes such as spatially varying selection and advantageous allele sweeps previously discussed may also contribute to reduced genetic variation at selected or linked loci. While these factors may all be contributing to the observed pattern it indicates that leading‐edge populations of current sardine range shifts may be genetically impoverished compared to their more established (central/southern) counterparts. This represents a noticeable contrast to anchovy (Petitgas et al., 2012) which has exhibited a similar expansion in the North Sea since the 1990's. Interestingly, Petitgas et al. (2012) propose that expansion of anchovy in the North Sea reflects an increase in abundance of a pre‐existing remnant population in the North Sea whereas for sardine the recent increase is considered a new colonisation following a period of absence in the area. As sardine is regarded as more mobile than anchovy (Bacha et al., 2014) these differences highlight that dispersal ability alone may not be a good predictor of range shift dynamics and leading‐edge richness and the need to understand of genotype‐environment interactions.

### 4.3 Implications for management

Management units containing multiple biological populations can lead to inaccurate descriptions of population‐specific abundances and productivity and may conceal the population declines (Kell et al., 2009). Conversely the use of management units that include only a portion of a larger population can present problems with understanding population stock dynamics and environmental linkages (Frisk et al., 2008). The lack of genetic differentiation between the Biscay and Channel/Celtic Sea samples suggests connectivity between these regions. While it might be tempting to suggest that sardine in these waters belong to a single panmictic population we advise caution. Firstly, as discussed previously contemporary connectivity may be overestimated due to historical gene flow. Second, even if levels of contemporary gene flow are sufficient to maintain genetic homogeneity this may not equate to enough dispersal to replenish stocks on timescales of interest to fishery managers (Hauser & Carvalho, 2008). Interestingly, the northern stock (ICES subarea 7) had historically been assessed together with the Bay of Biscay (area 8abd), until evidence of different growth rates, separate spawning grounds and the presence of all life stages in both areas led to them being split and managed separately (ICES, 2017, 2021).

Despite the overall weak genetic structure reported it is notable that both mtDNA and the outlier loci reported significant differentiation between the North Sea and South Biscay sample. Milano et al. (2014) and Westgaard et al. (2017) reported differentiation between Biscay and North Sea hake at an even smaller number of outlier SNPs (7 and 6, respectively) which they considered due to local adaptation, and which was later verified as also reflecting demographic independence (i.e. restricted dispersal) by Leone et al. (2019). Genetic differentiation between the Bay of Biscay and North Sea has been reported for multiple other species (Charrier et al., 2007; Huret et al., 2020; Leone et al., 2019). While demographic independence between North Sea and South Biscay would be incidentally aligned with the current separation of the central and northern stocks this needs to be confirmed and boundaries assessed. The lower level of variation for the North Sea sample also needs to be verified by analysis of samples collected from multiple sites and more recent dates. Relatively small losses of genetic variability may have irreversible consequences on the functional role of species within ecosystems and their long‐term viability. These small losses may represent unique genetic combinations that support the capacity of populations to adapt to contrasting environmental conditions or environmental change (Dann et al., 2013; Schindler et al., 2010). As genetic diversity is the raw material allowing species to adapt to new environmental conditions nascent fisheries activities in the North Sea must ensure that current low levels of genetic diversity are not further reduced through overharvesting as this will decrease the species adaptive potential (Bernatchez et al., 2017; Pinksy & Palumi, 2014) at its leading edge and potentially inhibit its expansion.

## AUTHOR CONTRIBUTIONS

**Niall J. McKeown:** Conceptualization (equal); data curation (lead); formal analysis (lead); methodology (lead); writing (lead). **Fabio Campanella:** Validation (equal); writing – review and editing (equal). **Joana F. Silva:** Writing – review and editing (equal). **Beatriz A. Roel:** Conceptualization (equal); funding acquisition (equal); project administration (equal); resources (equal). **Amy J. E. Healey:** Data curation (supporting); writing – review and editing (supporting). **Paul W. Shaw:** Resources (equal). **Jeroen van der Kooij:** Conceptualization (equal); funding acquisition (lead); writing – review and editing (supporting).

## CONFLICT OF INTEREST STATEMENT

The authors declare no known conflict of interest.

## Supporting information


Tables S1–S2.

Data S1.

## Data Availability

All mtDNA data are included with this submission. All nuclear data are freely available from the open access repository at https://pure.aber.ac.uk/.
