# Supplementary material for: Genomic analysis of NE Atlantic sardine (Sardina pilchardus) reveals reduced variation in a recently established North Sea population and directs reconsideration of management units
Source: Ecol Evol. 2024 Aug 1;14(8):e70101. doi: 10.1002/ece3.70101 (PMC11294039; doi:10.1002/ece3.70101)
Supplement: Supplementary file 1 — Tables S1–S2. [file ECE3-14-e70101-s001.docx]

Supplementary Table 1. Sample information and summary indices of genetic variation for the 9882 SNPs identified at MAF = 1% and genotyped in > 90% of individuals. Nuclear variation is described using number of polymorphic loci (Npoly), observed and expected heterozygosities (H_O_ and H_E_, respectively) and F_IS_ (with significant values in bold).

| Sample | Npoly | H_O_ | H_E_ | F_IS_ |
| --- | --- | --- | --- | --- |
| BB-S | 8138 | 0.142 | 0.162 | 0.122 |
| BB-N | 8001 | 0.142 | 0.159 | 0.101 |
| WC-F | 7328 | 0.142 | 0.165 | **0.142** |
| WC-ES2 | 8066 | 0.145 | 0.162 | 0.102 |
| WC-ES1 | 7973 | 0.146 | 0.161 | 0.094 |
| WC-EA | 8222 | 0.139 | 0.158 | 0.123 |
| CS-BC | 7760 | 0.134 | 0.159 | **0.154** |
| EC | 8212 | 0.139 | 0.158 | 0.111 |
| NS | 5680 | 0.110 | 0.152 | **0.274** |

Supplementary Table 2. Blow diagonal - pairwise F_ST_ values estimated from all 9882 SNPs genotyped in over 90% of individuals. All values were statistically non-significant. Above diagonal – pairwise Φ_ST_ estimated from mtDNA haplotype data with significant values in bold.

|  | BB-S | BB-N | WC-F | WC-ES2 | WC-ES1 | WC-EA | EC | CS-BC | NS |
| --- | --- | --- | --- | --- | --- | --- | --- | --- | --- |
| BB-S | - | -0.015 | -0.012 | -0.005 | -0.003 | -0.017 | -0.009 | -0.019 | **0.065** |
| BB-N | 0.002 | - | -0.013 | -0.015 | -0.013 | -0.014 | -0.021 | -0.012 | 0.021 |
| WC-F | 0.001 | 0.001 | - | -0.010 | -0.021 | -0.012 | -0.009 | -0.006 | 0.029 |
| WC-ES2 | 0.003 | 0.002 | -0.001 | - | -0.013 | -0.005 | -0.015 | -0.004 | 0.011 |
| WC-ES1 | 0.002 | 0.001 | 0.002 | 0.003 | - | -0.011 | -0.013 | -0.005 | 0.014 |
| WC-EA | 0.002 | 0.001 | -0.002 | 0.002 | 0.001 | - | -0.007 | -0.016 | 0.041 |
| EC | 0.004 | 0.002 | 0.002 | 0.002 | 0.003 | -0.003 | - | -0.014 | 0.014 |
| CS-BC | 0.003 | 0.002 | -0.001 | 0.002 | 0.002 | 0.001 | -0.003 | - | **0.049** |
| NS | -0.007 | -0.003 | -0.003 | -0.001 | -0.005 | 0.001 | 0.001 | -0.011 | - |
